# The Impact of Radiation Dose on Preoperative Neoadjuvant Chemoradiotherapy Effects for Patients with Locally Advanced Squamous Cell Esophageal Carcinoma: A Propensity Score-Matched Retrospective Study

**DOI:** 10.1155/2022/7581799

**Published:** 2022-10-15

**Authors:** Yanru Mu, Hui Wang, Tao He, Li Xu

**Affiliations:** Department of Radiotherapy, Bengang General Hospital of Health Industry Group, Benxi 117000, China

## Abstract

**Objective:**

To explore the impact of radiation dose on preoperative neoadjuvant chemoradiotherapy effects for patients with locally advanced squamous cell esophageal carcinoma (LASCEC) with long-term follow-up data.

**Methods:**

The patients with LASCEC received either low dose radiotherapy (50.4Gy/23f/1.8Gy) or a high dose (64.8Gy/25f/1.8Gy) followed by neoadjuvant chemotherapy preoperatively were included in this study. To balance potential bias, 1 : 1 propensity score matching (PSM) with a caliper of 0.1 was used. The two groups were compared in terms of radical resection, post-radiation adverse event rates, perioperative mortality, postoperative adverse event rates, overall survival (OS), local recurrence rate, and distant metastatic rate.

**Results:**

Forty-two patients were enrolled in this study, with 21 patients in each group after PSM. There was no difference in baseline characteristics between the two groups (all p >0.05). The rates of radical resection (71.4% vs 57.1%, P =0.334), perioperative mortality (9.5% vs 4.8%, P =0.549), and postoperative adverse event rates (76.2% vs 90.5%, P =0.410) did not differ significantly between the two groups. The 5-year OS rate was statistically higher in the group with a high dose (66.7% vs. 28.6%, P =0.013). Meanwhile, the local recurrence rate was statistically lower in the high dose group (14.3% vs 47.6%, P =0.019 for 3 years; 33.3% vs 66.6%, P =0.031 for 5 years). Moreover, the 3-year distant metastasis rate was statistically lower in the group with a high dose (9.5% vs 38.1%, P = 0.03).

**Conclusion:**

Patients with LASCEC may benefit from preoperative neoadjuvant chemoradiotherapy with a high radiation dosage (64.8Gy/25f/1.8Gy).

## 1. Introduction

Esophageal cancer is the ninth most frequent cancer globally and the seventh leading cause of cancer mortality [1]. Every year, China accounts for over half of all new esophageal cancer cases worldwide, and esophageal cancer ranks fifth and fourth in male morbidity and fatality in China, respectively [2]. Squamous cell carcinoma continues to be the most frequent histology in China, accounting for over 90% of all esophageal cancer cases [3].

Despite recent advances in endoscopy-related procedures, early detection of esophageal cancer remains difficult owing to the asymptomatic nature of the disease in its early stages. T2N0 lesions with any N+ tumors, cT3 or cT4a N, which are characterized as locally advanced squamous cell esophageal carcinoma (LASCEC) are found in a large percentage of esophageal cancer patients [4]. Esophagectomy is a key part of LASCEC therapy. However, esophagectomy alone is associated with recurrence and metastasis in 43.3 percent -5 0.0 percent of patients [5]. Neoadjuvant chemoradiotherapy (nCRT) combined surgery has been suggested as the first line treatment option for patients with LASECE to enhance their long-term prognosis [6–8]. However, following nCRT+surgery, the 5-year cumulative incidence of locoregional recurrence, distant recurrence, and total recurrence for LASCEC patients was 15.3%, 24.3%, and 32.2%, respectively, which was still not hopeful. Furthermore, a meta-analysis found that nCRT with surgery had a higher risk of overall postoperative mortality and treatment-related mortality in LASCEC than surgery alone [9]. As a result, a unique nCRT pattern is required to decrease morbidity and enhance prognosis in patients with LASCEC.

The protocols of nCRT have not been concluded yet. Variations in chemotherapy agents and radiation doses have been described. The most commonly used radiation doses were 41.4Gy [8] and 40Gy [10] in randomized controlled studies. Higher radiation doses have been explored, but there is presently no deterministic evidence to show a survival advantage [11]. Most studies found the treatment efficacy was not superior in patients who receive high radiation dose [12–14]. However, regarding LASCEC alone, the data is lacking. A recent study with Asian LASCEC patients included identified that high-dose radiotherapy with concurrent chemotherapy seems to be more effective with acceptable toxicity [11]. Therefore, the radiation dose for patients with LASCEC has not concluded yet. We conducted this study to explore the impact of the nCRT protocol with high radiation dose compared with standard radiation dose on patients with LASCEC with long term follow up data.

## 2. Materials and Methods

### 2.1. Patient Selection

From December 2011 to December 2016, the LASCEC patients who underwent nCRT in (Department of Radiotherapy Bengang Hospital of Liaoning Health Industry Group, Benxi City, Liaoning Province) were included in our study. All patients were diagnosed as SCEC by preoperative pathological biopsy. The tumor location and size were confirmed by swallowing gastrointestinal barium meal, and further imaging examinations such as CT and color ultrasound were performed to confirm that the tumors did not metastasize to supraclavicular lymph nodes and important organs in the chest and abdomen. Before therapy, all patients were staged according to the worldwide TNM staging criteria. The inclusion criteria were as follows: (1) patients aged 18 to 70 years who receive initial antitumor therapy; (2) international TNM standard staging of IIB or III; (3) thoracic esophageal cancer for which radical surgery is feasible; (4) no serious infection, anemia, normal coagulation, liver, kidney, heart functions and (5) Karnofsky functional status score ≥ 80. The exclusion criteria were as follows: (1) those who have received anti-tumor therapy before (2) hemorrhagic diseases are present or associated (3) previous surgery resulted in the inability to replace the esophagus with the stomach to reconstruct the digestive tract. Patients were separated into two groups based on their radiation doses: high (64.8Gy) and low (50.4Gy). Informed consent was obtained by all patients and their families. The research was authorized by our hospital's Medical Ethics Committee.

### 2.2. Neoadjuvant Chemoradiotherapy Treatment Protocols

For neoadjuvant chemotherapy, all patients were given paclitaxel 145 mg/m^2^ and cisplatin 75 mg/m^2^ on day 1^st^ and 21^st^. A total of 2 courses of chemotherapy were required.

Radiation and chemotherapy were carried out simultaneously. ELEKTA linear accelerator intensity modulated radiotherapy was used. Intensity modulated radiotherapy with ELEKTA linac was performed with 6MvX ray. The radiotherapy field was fixed with thermoplastic film and positioned with enhanced CT scans. All the radiation fields were involved. The detailed protocols were as follows: (1) Gross tumor volume (GTV): primary esophageal tumor and metastatic lymph nodes. (2) Clinical target area (involved field): the upper and lower part of the tumor 3 cm the esophagus, not exceeding the thoracic entrance and cardia, including para-esophageal lymphatic drainage area and metastatic lymph node drainage area; (3) Target area: expanding 8 mm on the basis of clinical target area. (4) Radiotherapy dose: image guided radiation therapy was adopted, routine segmentation, high dose group: 64.8Gy/25f/1.8Gy; low dose group: 50.4Gy/23f/1.8Gy, once a day, 4-5d/weeks. All radiotherapy was completed within 5 weeks.

### 2.3. Surgery Protocols

Four to five weeks after radiotherapy and chemotherapy, radical resection of esophageal cancer was performed after the recovery of bone marrow, liver and kidney function. With or without robot assistance, McKeown minimally invasive esophagectomy, comprising 2-field or 3-field lymphadenectomy and stomach reconstruction was performed. Patients with extensive adhesions or intraoperative hemorrhage were shifted to thoracotomy during the procedure. All surgeries were performed by competent surgeons who do more than 50 cases each year, ensuring surgical excellence. If a recurrence occurs after surgery, adjuvant chemoradiotherapy will be adopted according to the conditions of the patient.

### 2.4. Observation Indicators

The two groups were compared in terms of radical resection, post-radiation adverse event rates, perioperative mortality, postoperative adverse event rates, overall survival (OS), local recurrence rate, and distant metastasis rate.

The radical resection was defined as the R0 resection. The Radiation Therapy Oncology Group (RTOG) acute radiation injury grade standards were used to evaluate the post-radiation adverse event rates [9]. Severe complications were defined as RTOG classification grade III or above. The Clavien-Dindo classification was used to categorize postoperative complications, with Clavien-Dindo classification grade 3 being considered serious. Perioperative mortality was defined as patients died due to the complications of surgery within 30 days after surgery. Postoperative adverse events were defined as any complications related to surgery. Metachronous growth of a tumor in the remnant esophagus was characterized as a local recurrence. The advent of cancers outside the esophagus, including lymph metastasis and distant organ metastasis, was classified as distant metastasis. The OS was calculated as the time between the first operation and death or the final follow-up, including perioperative fatalities.

### 2.5. Statistical Analysis

On the basis of age, sex, tumor location, tumor size, and TNM staging, patients receiving high and low doses were propensity score matched at a 1 : 1 ratio. With a caliper of 0.1, a nearest-neighbor matching approach was applied. The chi-square test or Fisher's exact test was used to compare groups. Categorical data were reported as frequencies (percent) with total observations (n). Continuous data were reported as medians with interquartile ranges (IQR), and the Student's t or Mann–Whitney U tests were used to compare groups. The Kaplan-Meier technique was used to evaluate OS, local recurrence rate, and distant metastatic rates using log-rank comparisons. Furthermore, the OS, local recurrence rate, and distant metastasis rates were compared between the two groups at 1-, 2-, 3-, and 5-year follow-up. The SPSS program was used for all statistical analyses (version 20; IBM Corp, Somers, NY). P values of 0.05 were used to determine statistical significance.

## 3. Results

### 3.1. Baseline Characteristics

In our institution, 76 LASCEC patients got nCRT between December 2011 and December 2016 met the inclusion and exclusion criteria, with 22 patients receiving high dose radiation and 54 receiving low dose radiation. Following PSM, the final analysis contained 42 patients, including 21 individuals in each group.  Table 1 shows the baseline data of the two groups following PSM, with no significant differences in baseline features between the two groups. After PSM, the two groups were well balanced, with all standardized mean differences around 0.1.

### 3.2. The Effects of Radiation Dose on Short-Term Indicators

The radical resection rate was higher in the high dose group (71.4%) than the low dose group (57.1%). However, the difference was not significantly different (*χ*^2^ = 0.933, P =0.334). Similarly, the perioperative mortality was also higher in the high dose group (9.5%) than the low dose group (4.8%) with no significant difference observed (*χ*^2^ = 0.359, P =0.549) (Table 2).

After radiation, radiation esophagitis occurred in 19 patients (90.5%) in the high dose group and 17 patients (81.0%) in the low dose group, respectively. Radiation pneumonitis occurred in 10 patients (47.6%) and 11 patients (52.4%) in the high dose and low dose groups, respectively. Myelosuppression occurred in 9 patients (42.9%), including 3 severe cases (14.3%) in high dose group. Myelosuppression occurred in 8 patients (38.1%), including 2 severe cases (9.5%) in the low dose group. Moreover, gastrointestinal injury occurred in 12 patients (57.1%) and 10 patients (47.6%) in the high dose and low dose groups, respectively. In total, 18 (85.7%) patients experienced post-radiation adverse events in the high dose group and 3 of them were severe complication. 17 patients (81.0%) experienced post-radiation adverse events in the low dose group and 2 of them were severe complications. The post-radiation adverse event rates had no difference between the 2 groups (*χ*^2^ = 0.382, P = 0.537) and the post-radiation severe adverse event rates were also not significant difference between the 2 groups (*χ*^2^ = 0.227, P = 0.634) (Table 2).

After esophagectomy, in high dose group, 2 (9.5%) patients developed pulmonary infections; 2 (9.5%) patients developed chylothorax; 5 (23.8%) patients developed anastomotic fistulas, 6 (28.6%) patients developed anastomotic stenoses, and 1 patient developed cardiac complication (4.8%). In total, 14 (66.7%) patients developed postoperative adverse events in the high dose group, including 6 (28.6%) severe adverse events. In low dose group, 3 patients (14.3%) developed pulmonary infections, 4 patients (19%) developed chylothorax, 6 patients developed anastomotic fistulas (28.6%), 4 patients (19%) developed anastomotic stenoses, 2 patients developed cardiac complications (9.5%). In total, 14 (66.7%) patients developed postoperative adverse events in low dose group, including 6 (28.6%) severe adverse events. The postoperative adverse event rates had no difference between the 2 groups ((*χ*^2^ = 0.404, P = 0.525) and the postoperative severe adverse event rates were also not significant difference between the 2 groups (P = 1.000) (Table 2).

### 3.3. The Effects of Radiation Dose on Long-Term Follow-Up Outcomes

**Figure 1:**
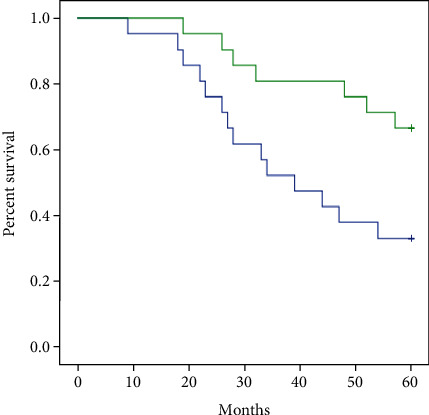
Kaplan-Meier survival curves depicting local recurrence rate among high dose group (**green line**) and low dose group (**blue line**). The curative effect of the study group is better than that of control group Log Rank = 5.528 P =0.019.

When the local recurrence rates of the two groups were compared, the high dose group was significantly lower than the low dose group (Log Rank = 5.528, P =0.019). At the follow up time points of 3 years (14.3% vs 47.6%, P =0.019) and 5 years (33.3% vs 66.7%, P =0.031), the local recurrence rates were significantly lower in the high dose group (Figure 1, Table 3).

**Figure 2:**
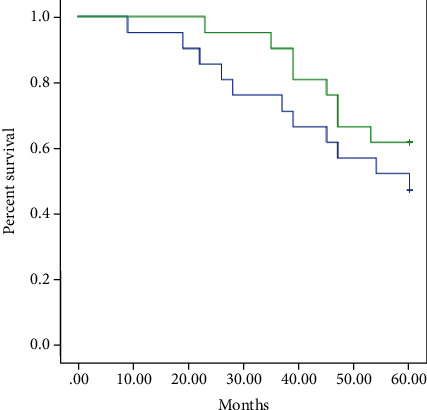
Kaplan-Meier survival curves depicting distant metastasis rate among high dose group (**Green line**) and low dose group (**blue line**). There is no difference between the study group and the control group Log Rank = 1.120 P = 0.290.

There was no significant difference in distant metastasis rates between the two groups (Log Rank = 1.120, P = 0.290). However, at the follo- up time point of - years, the distant metastasis rates were significantly lowerin the high dose group (38.1% vs 9.5%, P = =0.030) (Figure 2, Table 3).

**Figure 3:**
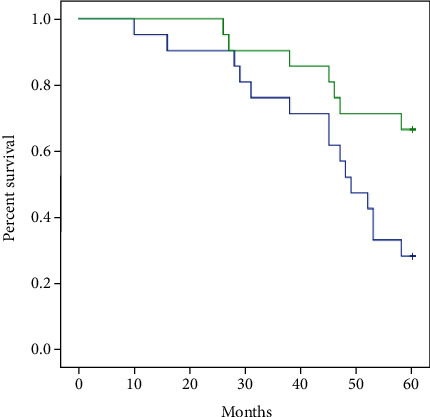
Kaplan-Meier survival curves depicting overall survival rate among high dose group **(green line**) and low dose group (**blue line**). The curative effect of the study group is better than that of control group Log Rank = 5.418 P = 0.020.

When comparing the two groups' OS rates, the high dosage group outperformed the low dose group (Log Rank = 5.418, P = 0.02). At the 5-year follow-up time point, the high dosage group had substantially improved OS rates than the low dose group (66.7% vs 28.6%, P =0.013) (Figure 3, Table 3).

## 4. Discussion

Surgical resection remains an important part of therapy for all resectable esophageal cancers. However, even following curative resection, surgery alone had poor long-term results, with 5-year survival rates seldom exceeding 30% [5, 15]. Some studies have shown that nCRT followed by surgery improves survival over surgery alone [8, 16, 17]. However, the radiation treatment dosage has not yet been determined.

Radiation dosage escalation for treating esophageal cancer should be researched further, according to NCCN recommendations, which prescribe a dose of 50 or 50.4 Gy for definitive concomitant chemoradiation [7]. The NCCN guidelines' recommendations are based on the findings of the RTOG 9405 trial [18]. In patients with Stages I–III squamous cell carcinoma or adenocarcinoma, this study assessed treatment responsiveness to concurrent chemoradiation utilizing 64.8 Gy against 50.4 Gy irradiation. However, this research found no evidence that a high dosage improved survival. Treatment-related mortality were more common in the high-dose group, and patients in this group had a poorer prognosis. However, the majority of fatalities in the high-dose group occurred in patients who got 50.4 Gy or less; hence, high-dose radiation may not be to blame for this group's higher mortality. Furthermore, the findings were largely focused on individuals who had been diagnosed with esophageal adenocarcinoma. In another study by Hulshof et al. [19], they concluded that the 3-year local progression-free survival (LPFS) was 70% in the standard dose (SD) arm versus 73% in the high dose (HD) arm (not significant). The LPFS for SCC and AC was 75% versus 79% and 61% versus 61% for SD and HD, respectively (not significant). The 3-year locoregional progression-free survival was 52% and 59% for the SD and HD arms, respectively. Although not fully significant, the HD arm had better treatment efficacy than the SD arm in numeric terms, especially the 3-year locoregional progression-free survival (P = 0.08). Extending the sample size may lead to different results. Therefore, do not based only on these studies, especially for patients with LASCEC. We conducted this propensity score-matched retrospective study and found that high-dose radiation offers a better long-term prognosis than low-dose radiotherapy.

The benefit of nCRT lied on the improved local control rate [11, 15, 20]. Given the significant local failure rates after 50 Gy [21] for patients who will not be surgical candidates following nCRT, high-dose radiation (together with concurrent chemotherapy) should be the best option for these patients. In certain people, surgery may be retained as a salvage option if there is residual disease following high-dose radiation. As a result, several hospitals are now using high-dose (60 Gy or greater) radiation with concomitant chemotherapy to treat LASCEC. The benefit of nCRT with high dose radiation therapy followed by surgery still need validation. Hence, we conducted this study to compare the impact of high dose and low dose radiation therapy (plus concurrent chemotherapy) followed by surgery with long-term follow-up data.

We noticed that 3 of 42 patients (7.1%) had perioperative deaths in our study. Although the death rate was lower than FFCD 9102 trials (9%) [12] and a randomized trial by Stahl et al. (11.3%) [21], the mortality rate cannot be ignored. Therefore, some studies concluded that definitive nCRT had comparable efficacy with cCRT followed by surgery with lower perioperative deaths [16]. According to the ESMO recommendations, nCRT followed by resection is the same as definitive nCRT. Esophagectomy should only be done in high-volume hospitals, according to the guidelines [22]. However, in medically fit patients, the dearth of recent randomized studies comparing definitive chemoradiation to induction chemoradiation followed by surgery provides a therapeutic conundrum. Therefore, the strategy that nCRT followed by surgery was still adopted in our center.

Our study also showed that high dose nCRT was not associated with a significant increase in postoperative complications. Most patients had fewer than grade 2 complications, and further treatment was not necessary. However, almost two-thirds of them experienced postoperative complications. The complication rate was unexpectedly high. The following are possible reasons: Surgeons may execute a difficult esophagectomy after nCRT, resulting in surgical difficulties and postoperative problems. Radiation, for example, may have a role in the development of an anastomotic leak and postoperative acute lung damage. More work is needed to address the high incidence of postoperative complications.

There were some limitations to our research that should be discussed. First, since the research was retroactive, we were unable to draw more conclusive conclusions. The tiny sample size was the second constraint. In our facility, individuals who got a high dosage of radiation were uncommon. Furthermore, patients were often moved from other referral facilities. Our medical system did not properly show their original medical information and follow-up data. The single institution series was severely constrained by these two reasons. To learn more about how high doses of radiation affect nCRT, we need more multicenter prospective studies with large sample sizes.

## 5. Conclusion

High-dose radiation combined with chemotherapy is a successful treatment for LASCEC with acceptable toxicity and a better long-term prognosis. To generate better evidence, a larger sample size and more follow-up will be necessary.

## Figures and Tables

**Table 1:** Comparison of clinical data between high dose group and low dose group after propensity score matching.

	Control group (N = 21)	Study group (N = 21)	Statistical test value	P
Gender (male/female)	11/10	14/7	0.889	0.346
Age (y) ± SD	66.33 ± 10.67	65.14 ± 10.79	0.360	0.721
Lesion site				
Upper thoracic segment	4	5	0.159	0.924
Middle thoracic segment	11	10		
Inferior thoracic segment	6	6		
Maximum diameter of lesion (cm) ± SD	4.29 ± 2.17	3.67 ± 2.44	0.869	0.390
TNM staging				
II	10	6	1.615	0.204
III	11	15		

SD: standard deviation.

**Table 2:** Comparison results of short-term indicators between high dose and low dose groups.

	High dose group (n = 21)	Low dose group (n = 21)	*χ* ^2^	P
Radical resections	15 (71.4%)	12 (57.1%)	0.933	0.334
Perioperative deaths	2 (9.5%)	1 (4.8%)	0.359	0.549
Post-radiation complications (n, %)	18 (85.7%)	17 (81.0%)	0.171	0.679
Radiation esophagitis	19 (90.5%)	17 (81.0%)	0.778	0.378
Radiation pneumonitis	10 (47.6%)	11 (52.4%)	0.095	0.758
Myelosuppression	9 (42.9%)	8 (38.1%)	0.364	0.546
Gastrointestinal injury	12 (57.1%)	10 (47.6%)	0.382	0.537
Severe post-radiation complications (n, %)	3 (14.3%)	2 (9.5%)	0.227	0.634
Postoperative complications (n, %)	14 (66.7%)	12 (57.1%)	0.404	0.525
Pulmonary infection	3 (14.3%)	2 (9.5%)	0.227	0.634
Chylothorax	4 (19%)	2 (9.5%)	0.778	0.378
Anastomotic fistula	6 (28.6%)	5 (23.8%)	0.123	0.726
Anastomotic stenosis	4 (19%)	6 (28.6%)	0.525	0.469
Cardiac complications	2 (9.5%)	1 (4.8%)	0.359	0.549
Severe postoperative complications (n, %)	6 (28.6%)	6 (28.6%)	0.000	1.00

**Table 3:** Comparison results of long-term follow up outcomes between high dose group and low dose group.

	Low dose group (n = 21)	High dose group (n = 21)	*χ* ^2^	P
Local recurrence rate, %				
Time points at 1-year	4.8%	0	0.000	1.000
Time points at 2-year	23.8%	4.8%	3.111	0.078
Time points at 3-year	47.6%	14.3%	5.459	0.019
Time points at 5-year	66.7%	33.3%	4.667	0.031
Distant metastasis rates, %				
Time points at 1-year	4.8%	0	0.000	1.000
Time points at 2-year	14.3%	4.8%	1.105	0.293
Time points at 3-year	38.1%	9.5%	4.725	0.030
Time points at 5-year	52.4%	38.1%	0.865	0.352
Overall survival rate, %				
Time points at 1-year	95.2%	100%	0.000	1.000
Time points at 2-year	90.5%	100%	0.525	0.469
Time points at 3-year	76.2%	90.5%	1.543	0.214
Time points at 5-year	28.6%	66.7%	6.109	0.013

## Data Availability

The data used to support the findings of this study are included within the article.
